# Birth Weight among Singletons Born to Foreign-Born Mothers in Taiwan: A Population-Based Birth Register Study

**DOI:** 10.2188/jea.JE20080096

**Published:** 2009-05-05

**Authors:** Yu-Ming Shen, Lai-Chu See, Sheue-Rong Lin

**Affiliations:** 1 Biostatistics Consulting Center, Department of Public Health, Chang Gung University, Taoyuan, Taiwan; 2 Director General Office, Public Health Bureau of Taoyuan County, Taoyuan, Taiwan

**Keywords:** birth weight, transnational marriage, foreign-born mothers

## Abstract

**Background:**

We compared the birth weight of newborns born to foreign-born mothers (FBMs) and Taiwan-born mothers (TBMs), using data from the 2005–2006 Taiwan Birth Registry of singleton live births.

**Methods:**

The Wilcox–Russell method, data restriction, and multiple linear regression were used to analyze the data. The rates of low birth weight (<2500 g) with 95% confidence intervals were computed for TBMs, and for each of the nationalities of FBMs.

**Results:**

The mean birth weight of newborns of FBMs was 3157 g, which was higher than that of newborns of TBMs (3109 g). On analysis using the Wilcox–Russell method, both the rate and residual proportion of low-birth-weight (LBW) births were lower among newborns of FBMs (4.1% and 1.1%, respectively) than among newborns of TBMs (5.9% and 1.7%, respectively). After adjusting for sex, mode of delivery, maternal age, smoking status, predisposing maternal risk factors, and condition during pregnancy, the newborns of FBMs weighed 72.9 g (95% CI, 68.8 g to 77.0 g) more than the newborns of TBMs. When data were restricted to mothers without any adverse conditions and adjusted for maternal age, the differences in birth weight between the 2 groups remained unchanged. The rates of LBW deliveries among FBMs in Taiwan were significantly lower than those in their respective countries of origin.

**Conclusions:**

In Taiwan, newborns of FBMs had a higher birth weight than those of TBMs, even after accounting for potential confounding factors, and had lower rates of LBW deliveries than did mothers in their respective countries of origin.

## INTRODUCTION

Many previous studies have shown that babies born to foreign-born mothers tend to have lower rates of low birth weight (LBW, birth weight <2500 g) than babies born to native-born mothers, despite the unfavorable socioeconomic status of the former.^1^^–^^5^ Is this evidence of a protective effect of foreign-born status in birth weight? The focus of this study was to determine if there are significant genetic, migration, and/or behavioral factors that explain the difference in birth weight of infants born to foreign-born mothers residing in Taiwan and those born to Taiwan-born mothers.

The literature indicates that the lower LBW rate among immigrants may be due to economic, noneconomic, and selection effects.^6^^,^^7^ However, Wilcox and Russell hypothesized that this LBW paradox may simply be an artifact that results from the fact that a small sample of foreign-born mothers tends to have a lower standard deviation of birth weight than the larger sample of native-born mothers.^8^^,^^9^ Ethnic differences in birth weight continue to be a subject of debate, and evidence from different settings is required to reach a definitive conclusion.

The Taiwan Birth Registry contains data on ethnic disparities in birth weight. In Taiwan, transnational marriages accounted for 20% of registered marriages in 2005.^10^ Maternal nationality has been recorded in the Taiwan Birth Registry since 2004 to assist in government allocation of resources to babies of foreign-born mothers (FBMs) and Taiwan-born mothers (TBMs).

Typically, transnational marriages in Taiwan are arrangements made by marriage brokers and frequently involve a Taiwanese man and a woman from Southeast Asia or mainland China.^11^ The main purpose of transnational marriages is to continue the bloodline of the family and to ensure management of household affairs.^12^ The Taiwanese men in transnational marriages are frequently of low socioeconomic status; some are physically or mentally handicapped.^11^^,^^12^ The foreign-born women often become pregnant or give birth shortly after immigrating to Taiwan, even though they may still be adjusting to their new environments.^11^^–^^13^ Therefore, it is reasonable to speculate that babies of FBMs start with disadvantages.

The National Health Insurance in Taiwan (NHIT) provides subsidized prenatal care to individuals in all socioeconomic classes. The percentage of the population covered by the NHIT increased from 89.5% in 1995, when NHIT was founded, to 99% in 2004.^14^ NHIT provides 10 free visits for prenatal care and labor.^15^ Regardless of the socioeconomic status of the family, the health care of the mothers and newborns during pregnancy and delivery is subsidized by the NHIT.

The Taiwan Birth Registry contains information on obstetrical diseases and the medical histories of expectant mothers, which allows us to compare the difference in birth weight between 2 birth groups with respect to predisposing conditions or the circumstances of the pregnancy. Unless the fetus suffers serious health problems during pregnancy, most expectant mothers will have prenatal care and give birth at the same medical facility.^15^ Hence, physicians or midwives are able to document the health status of the expectant mother from the beginning to the end of the pregnancy when they report the birth to the local health office.^16^

To address the birth weight paradox, we compared the birth weight of 2 groups of mothers (FBMs vs. TBMs), using data from the Taiwan Birth Registry for 2005–2006. The Wilcox–Russell method, restriction, and multiple linear regressions were used for data analysis. The LBW rates for TBMs and FBMs, in Taiwan and in their respective native countries,^17^ were also investigated.

## METHODS

Birth data were obtained from the 2005–2006 Taiwan Birth Registry, Bureau of Health Promotion, Department of Health, Taiwan. The Institutional Review Board of the Bureau of Health Promotion, Department of Health, Taiwan approved this study. Only singleton live births with a gestational age of at least 24 weeks were included in the study because induced abortion is legal in Taiwan before 24 weeks of gestational age and because the fetus has a better chance of survival outside the uterus after 24 weeks of gestation. We excluded records of birth weights that were aberrant for gestational age, as determined by expert clinical opinion and statistical analysis (proposed by Alexander et al^18^). Because there were only small numbers of babies born to mothers from developed countries—including Japan, Korea, South Africa, England, and USA—their records were excluded.

The Taiwan Birth Registry provides the following information:

(1) Newborn: sex, gestational age, birth weight, date and time of birth, plurality (single, twin, triplet, etc), health problems of the newborn, live birth or stillbirth.
(2) Demographic characteristics of parents: maternal and paternal nationality, dates of birth of the mother and father.
(3) Delivery: location of the delivery center, who delivered the baby (physician or midwife), and mode of delivery.
(4) Conditions during pregnancy: gestational hypertension, gestational diabetes, toxemia of pregnancy, cervical incompetence, polyhydramnios (amniotic fluid index (AFI) >24 cm or deepest amniotic fluid pool (DP) >8 cm) or oligohydramnios (AFI <5 cm or DP <1 cm).
(5) Predisposing maternal factors: anemia (hematocrit <30 or hemoglobin <10), history of preterm delivery (gestational age <37 weeks) or LBW baby (birth weight <2500 g), thalassemia, diabetes mellitus, cardiac disease, chronic hypertension, history of high-birth-weight delivery (birth weight >4000 g), syphilis, pulmonary disease, nephropathy, and rhesus isoimmunization.
(6) Adverse maternal behavior: cigarette smoking, alcohol drinking, and illicit drug use during pregnancy.

The chi-square test and independent *t* test were used to compare categorical and continuous data on maternal characteristics, univariately when appropriate. For birth weight, we used the method of Wilcox and Russell,^8^^,^^9^ which involves estimating the predominant normal birth weight distribution and the left-skewed residual percentage of small preterm babies in the distribution of birth weight. The predominant distribution closely corresponds to the birth weight distribution of term births (≥37 completed weeks of gestation). Wilcox and Russell^8^^,^^9^ demonstrated that in any large data set the empirical distribution of term births alone is almost purely normal, with a mean and standard deviation closely approximated by the predominant distribution of all births. However, the residual distribution comprises all births in the lower tail of the curve that fall outside the predominant distribution. In a typical population, 2% to 5% of births are in this residual distribution. Wilcox and Russell also maintained that virtually all births in the residual distribution are preterm births, but that not all preterm births are in the residual distribution.

Next, we followed a sequential modeling strategy, using a linear regression procedure, based on all eligible records, and restricted the data to include only mothers without any maternal adverse conditions. Regression models were stratified by nationality. Combined models were then examined for changes in mean birth weight between FBMs and TBMs, after adjusting for important maternal risk factors. LBW rates (%) with 95% confidence intervals (CI) were computed to enable international comparisons. The significance level of this study was 0.05. All analyses were performed using Statistical Analysis Software (SAS) version 9.1.

## RESULTS

There were 416 335 records in the 2005–2006 Taiwan Birth Registry. After excluding stillbirths (1.1%), non-singleton births (2.8%), gestational age of infants less than 24 weeks (0.6%), FBMs from developed countries (0.3%), and implausible birth weights for gestational age (0.6%), 399 551 records remained for analysis. A total of 50 049 (12.5%) were newborns of FBMs.

**Figure 1.**
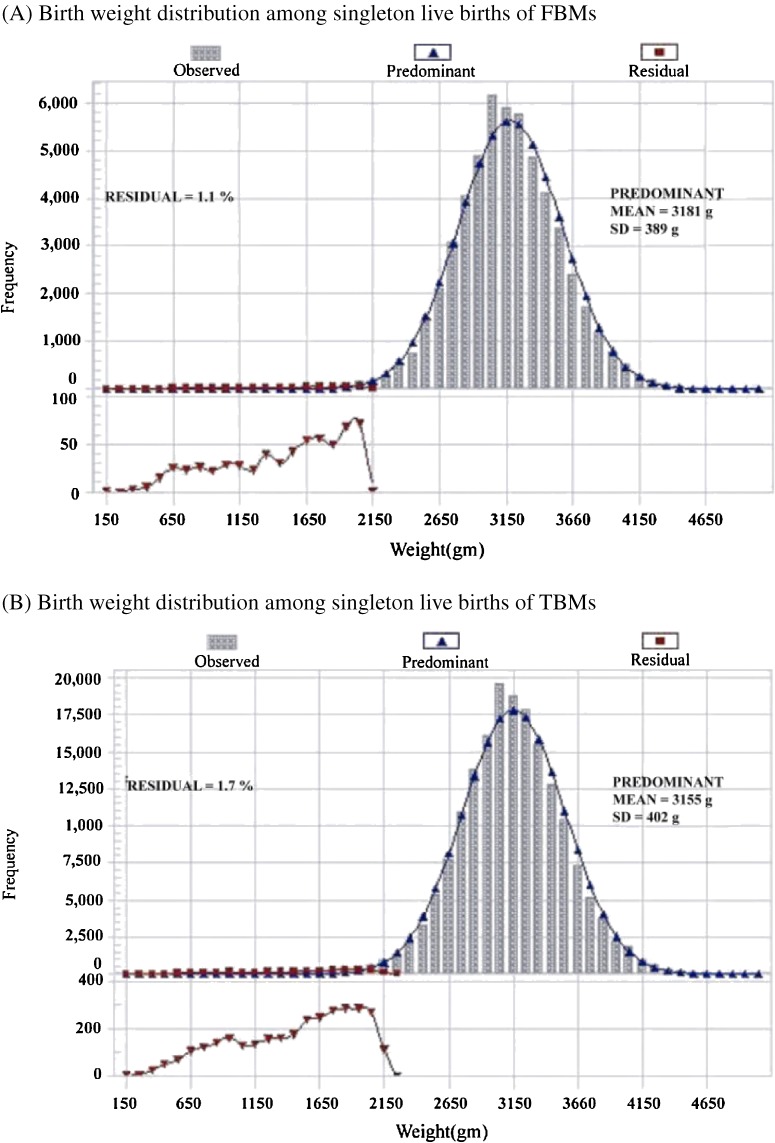
Birth weight distribution among singleton live births of foreign-born mothers (FBMs) and Taiwan-born mothers (TBMs), as determined using the Wilcox–Russell method (Taiwan Birth Registry 2005–2006)

The newborns of FBMs were heavier (mean, 3157 g) than those of TBMs (mean, 3110 g). The LBW rate among newborns of FBMs (4.1%) was significantly lower than among newborns of TBMs (5.9%). Male newborns were heavier than female newborns. Using the Wilcox–Russell method, the mean birth weight of the predominant distribution was 3181 g for newborns of FBMs and 3155 g for newborns of TBMs. The residual proportion was lower among the newborns of FBMs (1.1%) than among the newborns of TBMs (1.7%). (See Table 1, Figure 1A and 1B) In summary, newborns of foreign-born mothers displayed a more favorable birth weight distribution.

**Table 1.** Birth weight and low-birth-weight rate for singleton live births, by maternal nationality (Taiwan Birth Registry 2005–2006)

	Total (*n* = 399 551)	Newborns of FBMs (*n* = 50 049)	Newborns of TBMs (*n* = 349 502)	Difference or RR (95% CI)
Birth weight				
​ Mean ± SD (g)	3115.5 ± 434.2	3157.2 ± 415.1	3109.5 ± 436.5	47.7 (43.6–51.8)
​ LBW (<2500 g)	22 708 (5.7%)	2027 (4.1%)	20 681 (5.9%)	0.68 (0.65–0.72)
Sex				
​ Male	3159.8 ± 438.8	3201.8 ± 423.3	3153.8 ± 440.7	43.3 (37.8–48.8)
​ Female	3067.0 ± 423.7	3108.6 ± 400.2	3061.0 ± 426.7	43.4 (37.7–49.1)
Wilcox–Russell method				
​ Predominant distribution ​ ​ Mean ± SD (g)	3155 ± 398	3181 ± 389	3155 ± 402	
​ Residual proportion	1.6%	1.1%	1.7%	
​ Truncation point (g)	2200	2200	2200	

FBMs: Foreign-born mothers; TBMs: Taiwan-born mothers; RR: relative risk; CI: confidence interval; LBW: low birth weight.

**Figure 2.**
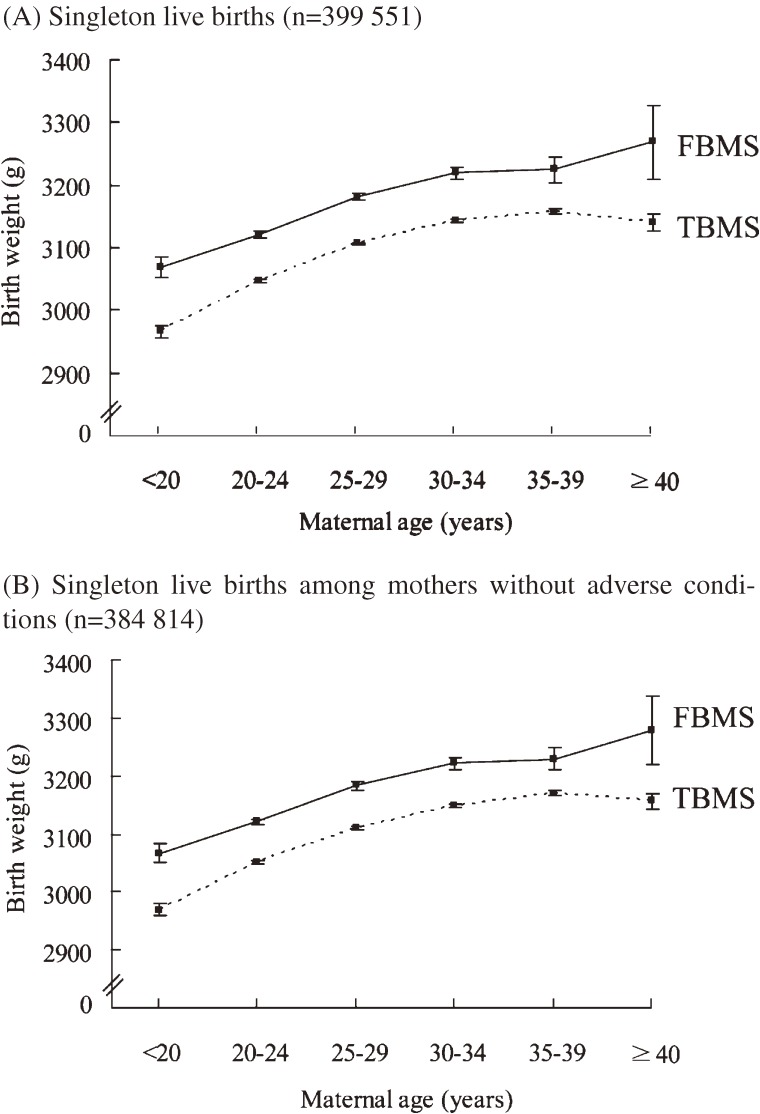
Mean birth weight and maternal age for singleton live births, by maternal nationality (Taiwan Birth Registry, 2005–2006)

The effect of maternal age on birth weight was analyzed. The newborns of FBMs consistently weighed more than newborns of TBMs, among all maternal age subgroups. However, a greater difference between FBMs and TBMs in the birth weights for newborns were seen among teenaged mothers and mothers older than 40 years (see Figure 2A ). When we restricted the analysis to mothers without any adverse conditions, a similar pattern of birth weight with respect to maternal age and maternal nationality was observed. (See Figure 2B)

In the comparison of the maternal characteristics between FBMs and TBMs, the characteristics of FBMs tended to be more favorable in terms of age, substance use history, predisposing maternal risk factors, and health condition during pregnancy, with the exception of the prevalence of syphilis. The rates of substance use among both groups were extremely low (See Table 2).

**Table 2.** Maternal characteristics by nationality (Taiwan Birth Registry 2005–2006)

	FBMs (*n* = 50 049)		TBMs (*n* = 349 502)		Difference or RR (95% CI) in FBMs
Maternal age (yrs)					
​ <20	2039	(4.07%)	8892	(2.54%)	1.71 (1.63–1.79)
​ 20–24	21 166	(42.29%)	53 516	(15.31%)	1.93 (1.91–1.95)
​ 25–29	16 932	(33.83%)	132 366	(37.87%)	reference
​ 30–34	7612	(15.21%)	112 049	(32.06%)	0.68 (0.66–0.69)
​ ≥35	2300	(4.60%)	42679	(12.21%)	0.49 (0.47–0.51)
​ Mean ± SD	26.15 ± 4.53		29.35 ± 4.80		3.20 (3.16–3.24)
Conditions during pregnancy					
​ Gestational hypertension	97	(0.19%)	2224	(0.64%)	0.30 (0.25–0.37)
​ Gestational diabetes	83	(0.17%)	2155	(0.62%)	0.27 (0.22–0.33)
​ Toxemia of pregnancy	93	(0.19%)	1704	(0.49%)	0.38 (0.31–0.47)
​ Polyhydramnios or ​ ​ oligohydramnios	75	(0.15%)	774	(0.22%)	0.68 (0.53–0.86)
​ Cervical incompetence	13	(0.03%)	212	(0.06%)	0.43 (0.24–0.75)
Predisposing maternal factors					
​ Anemia	286	(0.57%)	2132	(0.61%)	0.94 (0.83–1.06)
​ History of preterm or ​ ​ low-birth-weight delivery	70	(0.14%)	967	(0.28%)	0.51 (0.40–0.64)
​ Thalassemia	52	(0.10%)	725	(0.21%)	0.50 (0.38–0.66)
​ Diabetes mellitus	13	(0.03%)	505	(0.14%)	0.18 (0.10–0.31)
​ Cardiac disease	24	(0.05%)	423	(0.12%)	0.40 (0.26–0.60)
​ Chronic hypertension	7	(0.01%)	363	(0.10%)	0.13 (0.06–0.28)
​ History of high-birth-weight ​ ​ delivery	23	(0.05%)	249	(0.07%)	0.65 (0.42–0.99)
​ Syphilis	73	(0.15%)	109	(0.03%)	4.68 (3.48–6.29)
​ Pulmonary disease	6	(0.01%)	175	(0.05%)	0.24 (0.11–0.54)
​ Nephropathy	11	(0.02%)	107	(0.03%)	0.72 (0.39–1.34)
​ Rhesus isoimmunization	3	(0.01%)	35	(0.01%)	0.60 (0.18–1.95)
Substance use					
​ Cigarette smoking	3	(0.01%)	356	(0.10%)	0.06 (0.02–0.18)
​ Alcohol drinking	1	(0.0%)	62	(0.02%)	0.11 (0.02–0.81)
​ Illicit drug use	0	(0.0%)	130	(0.04%)	—
Other	140	(0.28%)	2116	(0.61%)	0.46 (0.39–0.55)

FBMs: foreign-born mothers; TBMs: Taiwan-born mothers; CI: confidence interval; RR: relative risk.

In models adjusted for sex, mode of delivery, predisposing maternal risk factors, and conditions during pregnancy, maternal age was significantly associated with birth weight in FBMs alone, in TBMs alone, and in FBMs and TBMs combined. Because of the small number of FBMs who smoked cigarettes, no significant difference in birth weight was seen in newborns of FBMs who smoked as compared to newborns whose FBMs who did not smoke. In TBMs, a significant difference in birth weight of −237.8 g (95% CI, −285.8 g to −189.8 g) was seen for TBMs who smoked cigarettes as compared to TBMs who did not smoke. With respect to syphilis status, a lower birth weight was seen in newborns whose mothers had syphilis as compared to newborns whose mothers did not have syphilis in FBMs alone, and in FBMs and TBMs combined. More importantly, the newborns of FBMs weighed 72.9 g (95% CI, 68.8 g to 77.0 g) more than the newborns of TBMs, after adjustment for predisposing maternal risk factors, conditions during pregnancy, newborn’s sex, mode of delivery, maternal age, cigarette smoking, and syphilis. When data were restricted to mothers without any adverse conditions, and adjusted for newborn’s sex, mode of delivery, and maternal age, the difference in birth weight between the 2 groups remained unchanged (see Table 3).

**Table 3.** Sequential multiple linear analyses of the differential effects of maternal nationality on birth weight (Taiwan Birth Registry 2005–2006)

	All eligible records*			Without any adverse maternal conditions		
		
	FBMs (*n* = 50 049)	TBMs (*n* = 349 502)	Both (*n* = 399 551)	FBMs (*n* = 49 059)	TBMs (*n* = 335 755)	Both (*n* = 384 814)
	Coefficient ± SE (95% CI)	Coefficient ± SE (95% CI)	Coefficient ± SE (95% CI)	Coefficient ± SE (95% CI)	Coefficient ± SE (95% CI)	Coefficient ± SE (95% CI)
Intercept	3309.7 ± 6.3	3242.8 ± 2.5	3242.1 ± 2.3	3310.1 ± 6.3	3242.4 ± 2.5	3241.7 ± 2.3
FBMs vs. TBMs			72.9 ± 2.1 (68.8, 77.0)			73.1 ± 2.1 (69.0, 77.2)
Maternal age						
​ <20	−110.1 ± 9.6 (−128.9, −91.3)	−138.1 ± 4.7 (−147.3, −128.8)	−133.0 ± 4.2 (−141.2, −124.8)	−112.0 ± 9.5 (−130.6, −93.3)	−138.3 ± 4.7 (−147.5, −129.1)	−133.4 ± 4.2 (−141.6, −125.2)
​ 20–24	−58.7 ± 4.2 (−66.9, −50.5)	−57.9 ± 2.2 (−62.3, −53.6)	−58.5 ± 1.9 (−62.5, −54.8)	−59.1 ± 4.2 (−67.2, −50.9)	−57.5 ± 2.2 (−61.8, −53.2)	−58.2 ± 1.92 (−62.9, −54.5)
​ 25–29	Reference	Reference	Reference	Reference	Reference	Reference
​ 30–34	35.9 ± 5.6 (24.9, 46.9)	36.7 ± 1.7 (33.4, 40.0)	36.9 ± 1.7 (33.3, 39.9)	35.8 ± 5.6 (24.8, 46.7)	38.0 ± 1.7 (34.7, 41.3)	37.8 ± 1.6 (34.9, 41.9)
​ ≥35	48.3 ± 9.1 (30.5, 66.1)	51.7 ± 2.4 (47.0, 56.4)	51.3 ± 2.3 (46.8, 55.8)	45.3 ± 9.2 (27.3, 63.3)	55.2 ± 2.4 (50.5, 59.9)	54.4 ± 2.3 (49.9, 58.9)
Cigarette Smoking	2.9 ± 235.0 (−457.7, 463.5)	−237.8 ± 24.5 (−285.8, −189.8)	−236.1 ± 24.3 (−283.7, −188.5)			
Syphilis	−118.0 ± 47.7 (−211.4, −24.5)	−69.4 ± 40.9 (−149.6, 10.8)	−87.3 ± 31.5 (−149.0, −25.6)			

FBMs: foreign-born mothers; TBMs: Taiwan-born mothers; SE: standard error; CI: confidence interval.

*Adjusted for sex, mode of delivery, gestational hypertension, gestational diabetes, toxemia of pregnancy, polyhydramnios or oligohydramnios, cervical incompetence, anemia, history of preterm or low-birth-weight delivery, thalassemia, diabetes mellitus, cardiac disease, chronic hypertension, history of high-birth-weight delivery, pulmonary disease, nephropathy, rhesus isoimmunization.

Because international studies of birth weight more commonly report their findings in terms of LBW rate than by mean birth weight,^17^ we list the LBW rate for the nations of origin of FBMs in Table 4. In our study, FBMs from mainland China had the lowest rate of LBW (2.9%), followed by those from Myanmar (3.4%), Thailand (3.6%), Vietnam (4.7%), Cambodia (5.0%), Indonesia (5.2%), and the Philippines (6.3%). The LBW rates among all live births (singleton and multiple) of FBMs in Taiwan were significantly lower than those of mothers in their respective countries of origin.

**Table 4.** Rate of low-birth-weight births for singleton and all live births, by maternal nationality (Taiwan Birth Registry 2005–2006)

	Taiwan Birth Registry 2005–2006		WHO estimate	
		
	Singleton (*n* = 399 551)	All births^†^ (*n* = 411 637)	All births^†^	
	LBW% (95% CI)	LBW% (95% CI)	LBW (%)	Year
Taiwan	5.92 (5.84–6.00)	7.58 (7.49–7.67)		
Foreign	4.05 (3.88–4.22)	5.39 (5.39–5.59)		
​ Philippines	6.26 (4.67–7.85)	7.73 (6.00–9.46)*	20%	2000
​ Indonesia	5.18 (4.51–5.85)	6.01 (5.29–6.73)*	9%	2002
​ Cambodia	5.01 (3.84–6.18)	6.39 (5.09–7.69)*	11%	2000
​ Vietnam	4.72 (4.44–5.00)	5.83 (5.53–6.13)*	9%	2000
​ Thailand	3.64 (2.24–5.04)	4.45 (2.91–5.98)*	9%	2001
​ Myanmar	3.40 (1.76–5.04)	4.77 (2.87–6.67)*	15%	2000
​ Mainland China	2.92 (2.69–3.15)	4.64 (4.35–4.93)*	6%	1998–99

Low birth weight (LBW) was defined as a birth weight <2500 g.

CI: confidence interval.

*Significant difference from country of birth, ie, 95% CI did not encompass rate of country of birth.

^†^All births, singleton and multiple.

## DISCUSSION

The Wilcox–Russell method, data restriction, and multiple linear regression analysis were used to examine data from the 2005–2006 Taiwan Birth Registry in attempt to explain the birth weight paradox. A variety of factors, including conditions during pregnancy, maternal factors, maternal age, substance abuse, syphilis status, prenatal care, and transnational marriage effects, were investigated.

In univariate analysis, we found that mean birth weight, LBW rate, and mean birth weight in both the predominant distribution and the residual population were more favorable among newborns of FBMs than among newborns of TBMs. After adjustment for sex, mode of delivery, maternal age, cigarette smoking, and adverse maternal conditions in multiple linear models, newborns of FBMs were heavier than newborns of TBMs. After adjustment for maternal age, newborn’s sex, and mode of delivery, data on mothers with no maternal risk factors still indicated that newborns of FBMs were heavier than those of TBMs. The LBW rates among all live births (singleton and multiple) of FBMs in Taiwan were also significantly lower than those of mothers in their countries of origin.

The hypothesis that differences in birth weight resulted from genetic dissimilarities due to race was the first attempt to explain the observed differences.^19^ However, this notion was contradicted by the findings of a study by David et al,^2^ which compared the distribution of birth weights among newborns of African-born black women, US-born white women, and US-born black women. They found that the birth weight distributions of newborns of African-born black women and US-born white women were similar but much higher than the birth weight distribution of the newborns of US-born black women. They concluded that genetic factors were unlikely to explain the difference in birth weight between foreign-born mothers and native-born mothers in the US population. In a study of births in Portugal, the mean birth weight of newborns of foreign-born African women was highest (3317 g), followed by newborns of Portuguese white mothers (3280 g) and newborns of Portuguese-born African mothers (3248 g).^4^ Guendelman et al reported that the newborns of Mexican-born and North African-born women residing in the United States, France, and Belgium had higher birth weights than newborns of native-born women of the same respective ethnic groups.^3^ In our study, we observed that the mean birth weight among newborns of FBMs was higher than that among newborns of TBMs. Also, the rates of LBW were lower among newborns of FBMs than among newborns in the mothers’ respective countries of origin. Based on a comparison of the birth weight of newborns of foreign-born and native-born women of the same ethnic group in the US, Portugal, France, Belgium, and Asia (this study), genetic factors appear unlikely to explain the paradox of birth weight. However, it should be pointed out that genetic information on the fetus is usually not available in data from the national birth registry or birth certificates. Further study may clarify the role of genetic factors on birth weight.

Conditions during pregnancy and predisposing maternal factors can affect the birth weight of newborns.^20^ In our study, the low rate of medical complications during pregnancy (eg, prevalences of anemia and gestational diabetes <1%) may have resulted from under-reporting and could bring our findings into question. Because FBMs and TBMs had the same number of prenatal visits due to health coverage by the NHIT,^21^ nondifferential misclassification of maternal exposure was expected. Because nondifferential misclassification introduces a bias toward the null value,^22^ a significant nonzero effect of maternal nationality on birth weight in our study suggests the possibility of an even stronger effect of maternal nationality on birth weight. After adjusting the data for adverse maternal conditions on multiple linear models, or restricting the data to mothers without any adverse factors, the birth weight in newborns of FBMs remained higher than that of newborns born to TBMs. We conclude that predisposing maternal factors and conditions during pregnancy do not explain the difference in the birth weights of the newborns.

The relation between maternal age and birth weight is well-known.^23^ In our study, birth weight increased with maternal age among the 2 maternal groups, with or without consideration of adverse maternal conditions. Moreover, we observed that the effect of maternal age on mean birth weight differed between foreign-born and Taiwan-born mothers: the effects of age were greater for the FBMs. In general, Taiwanese teenagers are students, which is not the case for FBMs. It is possible that teenaged TBMs delayed their utilization of prenatal care due either to a failure to recognize the symptoms of pregnancy, denial of the pregnancy, fear of their parents’ response to the pregnancy, lack of financial resources,^24^ conflict with school enrollment,^25^ or significant substance abuse.^26^ Delay in prenatal care among teenaged mothers might therefore account for the lower birth weight among TBMs. For the mothers aged over 40 years, parity may partially explain the differences in birth weight between the 2 birth groups. Unfortunately, parity is not recorded in the Taiwan Birth Registry. Many studies reported that birth weight is higher among women of higher parity.^27^ Prior study has revealed a marked increase in the fertility rate among FBMs aged between 15 and 29, whereas TBMs typically have children later in life.^28^ Moreover, because the marriage age for women in Taiwan is increasing,^29^ they may be having their first babies later in life. Hence, it is possible that the parity of TBMs aged over 40 years was lower than that of FBMs.

Maternal cigarette smoking has been consistently associated with reduced birth weight among a wide range of populations.^4^^,^^30^^,^^31^ The rate of cigarette smoking among FBMs in Taiwan (0.01%) was lower than rates reported among foreign-born mothers in other countries (6.7% for whites, 1.5% for blacks, 1.4% for Asians, and 1.6% for Hispanics).^5^ In addition, we observed extremely low rates of illicit drug use and alcohol drinking among FBMs in our study. The effects of healthy behaviors that women bring from their countries of origin have been reported.^32^ The low rates of use of addictive substances such as tobacco, alcohol, and illicit drugs among FBMs support the hypothesis that behavioral risk factors contribute to the birth weight advantage of newborns of FBMs.^32^

However, not all the conditions for FBMs are favorable. In this study, FBMs were more likely to have had syphilis than were TBMs. Maternal syphilis has been associated with a high risk of adverse pregnancy outcomes.^33^ The effect of maternal syphilis on birth weight was −87.3 g (95% CI, −149.0 g to −25.6 g). Although the maternal syphilis rate for FBMs (0.15%) was higher than that for TBMs (0.03%), the rates for both groups were much lower than those found in most underdeveloped countries.^33^^–^^35^ The maternal syphilis rates for FBMs were lower than those in their respective countries of origin. Prenatal treatment of maternal syphilis among FBMs, or the presence of a selection effect (FBMs have to a pass physical examination in order to immigrate to Taiwan), may explain the low rate of maternal syphilis in our study.

Prenatal care is crucial for reducing the risks of adverse maternal and infant health outcomes.^36^ In Taiwan, Hung et al showed that FBMs have the same numbers of prenatal visits as TBMs.^21^ Hence, the free prenatal care service provided by the NHIT may contribute to the higher birth weight of newborns of FBMs, as compared to the birth weights in their respective countries of origin (Table 4).

The selectivity of the migration stream has also been hypothesized to contribute to the higher birth weight of newborns of foreign-born mothers.^6^^,^^37^ In Taiwan, many FBMs were selected by marriage brokers and women entering brokered transnational marriages are required to pass physical examinations by the Ministry of Foreign Affairs, Taiwan. Finally, immigration to Taiwan may help to create improved standards of living that may influence birth weight.^38^

One strength of the Taiwan Birth Registry is the completeness of the data. In Taiwan, physicians or midwives who deliver a birth are responsible for completing a birth certificate and are required to report a birth within 7 days to the local office of public health. In addition, officers from municipal and county departments of health investigate birth registrations in different magistracies. For babies who were not delivered at a hospital, clinic, or by a midwife (0.01%), parents are responsible for reporting the birth to the office of Residency Administration. The community head and a police officer of the magistracy together complete the form of the birth investigation certificate after an inquiry on the birth. The newborn’s family will then take this birth investigation certificate to complete the process of birth registration. This comprehensive system of birth registration has been in place for many years. Therefore, we believe that there are few missing data in the Taiwan Birth Registry.^39^

It is unclear why the proportion of babies born to FBMs (12.5%) differed from the percentage of transnational marriages (20%).^10^ One possibility is that TBMs gave births several years after marriage^28^ and/or gave birth to more children than did FBMs. This requires further investigation.

The present research possesses several limitations due to the absence of important data, which are not collected for the Taiwan Birth Registry. These include data related to birth weight, including prenatal care,^36^ maternal size,^40^ parental education,^5^ socioeconomic status,^41^ social support,^42^ occupational stress,^43^ and nutrition.^44^ Second, although a large proportion of FBMs came to Taiwan via marriage trade, some did not. Third, a small portion of newborns in TBMs may be from second-generation transnational marriages because translational marriage in Taiwan became common after 1987.
